# The Uses of Reason in Times of Technical Mediation

**DOI:** 10.1007/s10699-015-9443-x

**Published:** 2015-10-29

**Authors:** Steven Dorrestijn

**Affiliations:** Hospitality Business School, Saxion University of Applied Sciences, Handelskade 75, Postbus 501, 7400 AM Deventer, Netherlands

**Keywords:** Technical mediation, Ethics, Foucault, Fourfold of subjectivation, Uses of reason

## Abstract

The art of living idiom suits well a practice-oriented approach in ethics of technology. But what remains or becomes of the functioning and use of reason in ethics? In reaction to the comments by Huijer this reply elaborates in more detail how Foucault’s art of living can be adapted for a critical contemporary ethics of technology. And the aesthetic-political rationality in Foucault’s ethics is compared with Wellner’s suggestions of holding on to the notion of code but with a new meaning. Foucault’s fourfold scheme of subjectivation and a distinction of “below and above reason” structure the argument.

This text supplements my paper on ‘The Care of Our Hybrid Selves: Ethics in Times of Technical Mediation,’ in reply to the two insightful commentaries by Galit Wellner and by Marli Huijer. My historical overview in three stages of philosophical analysis of technology with three different figures of technical mediation raised no pressing questions. The commentaries both focus mainly on the second part, which proposed an ethics of technology inspired by Michel Foucault’s ideas about the arts of living. Huijer wonders if I do not use Foucault’s work in an uncritical way when transferring his ideas from classical Greece and Rome to our days, and from the domain of sexuality to technology. Wellner suggests Self Determination Theory as an approach for giving more content to the care of our hybrid selves, which is necessary as she considers Foucault’s work effective for critical diagnosis but less helpful for new solutions.

Here, I will focus more extensively on the goals and the rationality of an ethics of technology, which I think is the main target of the comments. My initial paper, however, rather focused on the problematization of ourselves as hybrid beings and on ethical practices of self-care for coping with hybridization. I just implicitly made use of Foucault’s fourfold scheme for studying ethics, which was introduced in our discussion by Marli Huijer, when she mentioned four dimensions of the moral experience: ontology, deontology, ascetics, teleology. In my paper I chose to bring up only the simpler version that Foucault uses simultaneously, and which is referred to by Wellner, namely of a code-based ethics versus a practical arts of living. Elsewhere I extensively dealt with the fourfold (Dorrestijn 2006, 50–59, 2012b), even using it as the backbone structure of my Ph.D. thesis (Dorrestijn 2012a, 58–60). I find it a fascinating part of Foucault’s work and I am happy to discuss it in some more detail. In the course of doing so, I will outline how I want to proceed with this scheme myself. My ultimate interest is an understanding of human existence as simultaneously conditioned from “below” (being part of the world, mediated by technologies) as well as aspiring towards shared rationality and values “above”. In reaction to the comments I will now elaborate on the shift in ethics from codes to practice in terms of Foucault’s fourfold and conclude by discussing what remains or what may become of the use of reason.

## Four Dimensions of Subjectivation

The simpler scheme that Foucault uses in his work on ethics is a distinction between code-based ethics and ethics of subjectivation. In modern moral theory the universal rational law is central and humans are assumed to be rational subjects complying with the exigencies of the moral law. Foucault sees a more general meaning of ethics in the tradition of the practical art of living. Here the subject is not a precondition, rather the constitution of the moral subject itself is the center part of ethics. Foucault sees subjectivation as the more general meaning of the term ethics. The genealogy of the subject, of ethics in the sense of subjectivation became the project of Foucault’s later work. His genealogy of ethics reveals how in the course of history different understandings of the subject and different experiences of being a subject have prevailed.

In structuring this genealogy, Foucault introduces the fourfold scheme of subjectivation and investigates how different ethical systems differ along these dimensions. The scheme comprises: (1) ethical substance, (2) mode of subjection, (3) ethical elaboration, and (4) teleology (see Foucault 1992, 25–32; and 2000, 262–269). Huijer speaks of: (1) ontology, (2) deontology, (3) ascetics, (4) teleology. In Foucault’s work there is variation in the use of terms. That is partly a question of definition, but also partly of content: beyond the titles themselves, the dimensions of subjectivation have a different content in different times, different cultures, or for different persons.

While Foucault does nowhere explain the origins of his scheme, Gilles Deleuze (1988, 86) has noticed the analogy with Aristotle’s fourfold of material, formal, efficient and teleological causation. The link seems indeed undeniable, and indicates an alternative way of coming to grips with the scheme. Subjectivation appears as self-causation, with four kinds of causes, or constitutive facets: (1) the materiality of the self, (2) the model, or principle one recognizes oneself subject of, (3) the work or practices of self-formation, (4) the state of being that the care of oneself strives to achieve.

## A Critical Application of Foucault’s Fourfold

The question is indeed, as Huijer brings to the fore, if and how this scheme that Foucault developed to analyze the ancients’ dealing with sexuality can be applied to the integration of technologies in our lives today. Well, I think that it can be used as a tool for problematization and reflection. Take for example the *ethical substance* and its employment in Foucault’s research. Huijer remarks she will focus on the Greek and Latin problematization of “desire”, while she also refers to “sexuality” and “*aphrodisia*” (acts of pleasure). What I take as the important insight from Foucault is that these concepts do not mean the same, but point at different conceptions of the self (here in relation to “sexuality”) in different times and cultures. In the age of Freud (as I would phrase it) the self is conceived of as drum full of sexual desires which tend to burst out and therefore need to be cultivated. The Greeks, by contrast, knew no such *desire*; their concern focused not on the possibility of *controlling one’s desires*, but on how to make “use of pleasures*”* in a way that earns respect.

The criterion of earning respect brings us to the aspect of the *mode of subjection*. Huijer refers to that aspect as “deontology” and as “one ought”. Both these terms are taken from the vocabulary of code-based ethics. To me the originality of Foucault’s research is that he considers whether a call to engage in ethics can take a form that is at variance with moral duty. Foucault’s finding is that in ancient ethics the principle that one avowed oneself subject to was *aesthetical* and/or *political* rather than *moral* in the modern sense. The *reason* to fashion one’s sexual behavior was not a moral codex, but commitment to living a good, beautiful, successful, respectable life. These latter criteria do not sound *moral* to moderns ears like ours (or of our ancestors of the past few centuries). An important difference is that a universal rational ground is missing, since what counts as beautiful and respectable is dependent of the opinion and judgement of peers. In this light I think it is suitable that Foucault speaks of a politico-aesthetical mode of subjection in ancient ethics. The fascinating question this raises is whether such a different functioning or use of reason in ethics is indeed happening and desirable.

In the same way one could also discuss the e*thical elaboration* (ascetics) and *teleology*. There is not one correct way of using the fourfold scheme, but the scheme encourages reflection and discussion about facts and interpretations of ethical practice. This is the way I like to see the application of Foucault’s work on ethics for research today. I do not intend to suggest some “art of living after Foucault” as a ready-made ethics for today. Rather I have adopted from Foucault a scheme for comparing different historical ethical systems, that I also wish to apply for critically reflecting on ethics today. How would we ourselves understand the substance, subjection mode, elaboration and telos of subjectivation processes today, in the domain of sexuality, but also in other domains such as the integration of technologies in our lives?

## The Fourfold and Technology

Such a use of Foucault’s work on the art of living in principle encourages rather than discourages critical reflection and discussion. How does that work out for ethics in times of technical mediation? Let me explicate my ambivalent stance to technology. I tried to distance myself from an approach to technical mediation *as a theory* by Verbeek and to lesser degree Latour. Their theories appear to yield a temperament of reassurance about current developments rather than a critical attitude. The focus on historical and empirical detail is the good thing about their approaches. But the warning call attitude of classical philosophy of technology need and should not be swept away. The goal of my elaboration of technical mediation in historical perspective was to acknowledge earlier critiques. A “meticulous scrutiny” (Huijer) of the effects of technology is definitely my goal. Indeed that is why I consider the technically mediated, or hybrid self as the *ethical substance* in terms of Foucault’s scheme. And surely there are technical developments that I would rather reject than embrace.

My critique of Verbeek and Latour, that they theorize technical mediation as an *answer* to ethics instead of as a *problem*, is precisely at this point linked to Foucault’s scheme. An interpretation in Foucault’s scheme of Latour’s “provisory” claim that morality becomes delegated, yields the conclusion that the moral law as *mode of subjection* is replaced by “the rule of technology”. This is indeed how Verbeek uses Foucault’s scheme himself (unintendedly, too hastily, I presume). Verbeek explicitly employs Foucault’s fourfold in relation to technology, identifying the mediation of behavior by technology as the mode of subjection: the moral code replaced by “material codes” (Verbeek 2011, 83). Although a further comparison concerning the use of Foucault’s subjectivation scheme is a good way to point out the important divergences of Verbeek’s approach from mine, there is no room for it here (see Dorrestijn 2012). Instead I will continue by discussing how I do think the mode of subjection should be seen.

## The Uses of Reason

How are we called upon today for rejecting, accepting or transforming technologies? What reasons or values do we acknowledge? Does Foucault’s shift to a practical art of living and away from codes and their rational ground mean that the latter play no role anymore? This is a valid question, a frequent comment on Foucault’s work. Both Huijer and Wellner mention this.

Wellner gives an interesting twist to this point. She argues that the idea of an ethical codex should not be abandoned, but codes should be “charged with a new meaning”. I think this is not in conflict with Foucault’s approach. Foucault explores an aesthetical and/or political form of reason, which does not render a universal ethical codex, but does definitely also not mean abandoning rationality. It seems that what I would term a *different use of reason* comes close to Wellner’s *new meaning of codes*.

In addition Wellner argues, with Self Determination Theory, for a developmental approach to the self. I agree that such an approach allows for a combination with an ethics of technology after Foucault. Moreover I am interested in the developmental approach to rationality and the mind which this kind of psychological research can bring to ethics.

## Human Being Below and Above Reason

Foucault’s work is greatly inspiring for a practical turn in the ethics of technology. For me this brought, firstly, a focus on the human being as rooted in and mediated by the world and artifacts, as well as on ethical practices instead of codes. This covers the *substance* and the *elaboration* in Foucault’s ethical fourfold. After this practical turn I think it is a hugely interesting project to explore alternative functions and uses of reason, beyond the form of reasoning of fully objective, universal, and morally exigent certainties. Can reason be inventive and creative, goal-oriented without a determined goal, and never complete, but emergent and evolving? This would cover in addition the *subjection mode* and *teleology* of Foucault’s scheme.

Modern rationalistic ethics had difficulties to understand how the required free subject could actually exist as well as achieve the rational foundation of ethics that it believed in. Rational man has loose ends downwards as well as upwards. Technical mediation research helps to understand ourselves as hybrid beings (below reason), but the ethics of technical mediation should also explore new vocabularies, maybe new uses of reason to deal with this awareness (above reason). For such a reshuffling of the subjectivation scheme I find inspiration in Foucault’s discussion of Kant’s anthropology (Foucault 2008). I think this kind of approach to *the human being below and above reason* could satisfy Wellner’s posthuman as well as Huijer’s critical orientation.
